# Editorial: The potential role of gut microbiome in animal gut-linked diseases

**DOI:** 10.3389/fmicb.2023.1179481

**Published:** 2023-03-28

**Authors:** Hui Zhang, Mujeeb Ur Rehman, Yung-Fu Chang, Tang Zhaoxin

**Affiliations:** ^1^College of Veterinary Medicine, South China Agricultural University, Guangzhou, China; ^2^Directorate Planning and Development, Livestock and Dairy Development Department Balochistan, Quetta, Pakistan; ^3^State Key Laboratory of Marine Resource Utilization in South China Sea, College of Oceanology, Hainan University, Haikou, Hainan, China; ^4^Cornell University Ithaca, Ithaca, NY, United States

**Keywords:** gut microbiota, intestinal immunity, gastrointestinal diseases, metagenomics, probiotics

The animal gastrointestinal tracts contain trillions of microorganisms, which play critical roles in immune system maturation, intestinal epithelium differentiation and nutrient absorption and metabolism (Belkaid and Hand, 2014). The gut contains more than 10^14^ microorganisms, including bacteria, fungi, and viruses, which interact in a synergistic or antagonistic relationship to maintain a stable intestinal environment and function (Zheng et al., 2020). Stabilized gut microbiota has been demonstrated to be a prerequisite for the intestine to perform various complicated physiological processes, but gut microbial dysbiosis may cause multiple gastrointestinal diseases, including diarrhea, stomachache, and colitis. Moreover, the effects of the gut microbial community extend beyond the gastrointestinal system and can cause other systemic diseases. Similarly, several studies have reported the associations between diseases status and gut microbiota, in order to improve our knowledge on microbiome and host interactions and to develop an effective approach to rehabilitate perturbed animal and human microbial ecosystems.

The gut microbial alternations in animal gastrointestinal system or the differences in gut microbiome composition and function have been associated with a variety of diseases ranging from metabolic conditions and gastrointestinal inflammatory to colitis, and respiratory illnesses. In this area of research, Zhang et al. have conducted a bibliometric analysis of publications in the field of intestinal flora and ulcerative colitis research in the past 10 years, which summarizes current knowledge regarding the global research trends in intestinal flora and ulcerative colitis. On the other hands, to understand the microbial composition of the entire gut and to provide insights on how to improve the overall health and productivity of the animals. Chang et al. have updated our knowledge on the structure and function of the intestinal microbiota at different growth and developmental stages of Tibetan pigs, which plays an important role in their immune performance.

Currently, metagenomic analysis and high-throughput sequencing have been used for investigating gut microbial alterations in several diseases that are considered to be linked with gut microbes. Gut microbial comparison and analysis have the potential to benefit the understanding of the pathogenesis of various animal gut-linked diseases and the development of corresponding strategies to decrease the collateral damages. Hang et al. have briefly analyzed the influence of Shugan Decoction (SGD) on intestinal microbiota and fecal metabolites in diarrhea predominant irritable bowel syndrome (IBS-D) rats by multiple omics techniques, including metagenomic sequencing and metabolomics. The authors have shown that how SGD can regulate specific intestinal microbiota and some metabolic pathways, which may explain its effect of alleviating visceral hypersensitivity and abnormal intestinal motility in WAS-induced IBS-D rats. Similarly, Wang L. et al. have updated our knowledge by utilizing high-throughput sequencing to analyze the intestinal flora of Weining cattle, Angus cattle, and diarrheal Angus cattle. The authors have revealed the potential bacteria associated with diarrhea for the subsequent treatment of diarrhea in Angus cattle. In order to better understand the relationship between intestinal flora and health, the significant changes in the type and proportion of bacteria have been explored and explained that how diarrhea not only directly modifies the diversity and abundance of gut microbiota but also indirectly affects some functional bacteria.

The role of gut microbial regulation in the prevention and treatment of animal diseases, such as by fecal bacteria transplantation, probiotic supplementation and other means is one of the hot Research Topic these days. In the same direction, Lin et al. have conducted a study on Echinacea exert to confirm its influence of intestinal flora in immunosuppressed ducks. The authors concluded that Echinacea extract can improve the development of immunosuppressed ducks by modulating the intestinal immune function and by increasing the abundance of beneficial bacterial genera in the intestine in birds. Similarly, prenatal and early postnatal development are known to influence future health, in the same area of research, Wang H. et al. have performed a surrogate fostering experiment in mice to examine the relationship between the metabolic markers associated to insulin resistance and the composition of the gut microbiota. The authors findings revealed that alterations in the early growth environment may prevent fetal-programmed glucose metabolic disorder *via* modulation of the microbiota-gut-brain axis.

In conclusion, this Research Topic provided diverse knowledge on the role of gut microbiota and animal intestinal diseases occurrence using multidisciplinary approaches combining multi-omics techniques. However, there is still a lot of research gap to understand the importance and role of gut microbial regulation in the prevention and treatment of animal diseases. Thus, future research should be emphasized on the factors contributing to prevent or occur gut microbiota inked diseases in animals.

## Author contributions

All authors listed have made a substantial, direct, and intellectual contribution to the work and approved it for publication.
